# Skeletal muscle myosin heavy chain expression and 3D capillary network changes in streptozotocin-induced diabetic female mice

**DOI:** 10.17305/bb.2023.9843

**Published:** 2024-06-01

**Authors:** Nejc Umek, Luka Pušnik, Chiedozie Kenneth Ugwoke, Žiga Šink, Simon Horvat, Jiří Janáček, Erika Cvetko

**Affiliations:** 1 Institute of Anatomy, Faculty of Medicine, University of Ljubljana, Ljubljana, Slovenia; 2 Department of Animal Science, Biotechnical Faculty, University of Ljubljana, Ljubljana, Slovenia; 3 Laboratory of Biomathematics, Institute of Physiology of the Czech Academy of Sciences, Prague, Czech Republic

**Keywords:** Type 1 diabetes mellitus (T1DM), skeletal muscle, myosin heavy chain (MyHC) isoforms, capillary network analysis, muscle fiber composition, three-dimensional (3D) image analysis

## Abstract

It is not well-understood how type 1 diabetes (T1DM) affects skeletal muscle histological phenotype, particularly capillarisation. This study aimed to analyze skeletal muscle myosin heavy chain (MyHC) fibre type changes and 3D capillary network characteristics in experimental T1DM mice. Female C57BL/6J-OlaHsd mice were categorized into streptozotocin (STZ)-induced diabetic (*n* ═ 12) and age-matched non-diabetic controls (*n* ═ 12). The muscle fibre phenotype of the soleus, gluteus maximus, and gastrocnemius muscles were characterized based on the expression of MyHC isoforms, while capillaries of the gluteus maximus were assessed with immunofluorescence staining, confocal laser microscopy and 3D image analysis. STZ-induced diabetic mice exhibited elevated glucose levels, reduced body weight, and prolonged thermal latency, verifying the T1DM phenotype. In both T1DM and non-diabetic mice, the gluteus maximus and gastrocnemius muscles predominantly expressed fast-twitch type 2b fibers, with no significant differences noted. However, the soleus muscle in non-diabetic mice had a greater proportion of type 2a fibers and comparable type 1 fiber densities (26.2 **±** 14.6% vs 21.9 **±** 13.5%) relative to diabetic mice. T1DM mice showed reduced fiber diameters (*P* ═ 0.026), and the 3D capillary network analysis indicated a higher capillary length per muscle volume in the gluteus maximus of diabetic mice compared to controls (*P* < 0.05). Overall, T1DM induced significant changes in the skeletal muscle, including shifts in MyHC fibre types, decreased fibre diameters, and increased relative capillarisation, possibly due to muscle fibre atrophy. Our findings emphasize the superior detail provided by the 3D analytical method for characterizing skeletal muscle capillary architecture, highlighting caution in interpreting 2D data for capillary changes in T1DM.

## Introduction

Diabetes mellitus (DM) encompasses a range of metabolic disorders that disrupt the regulation of glucose levels in the body, leading to sustained high blood glucose levels [1]. Type 1 DM (T1DM) is one of the two common forms of DM. In contrast to the more prevalent type 2 DM classically associated with the development of insulin resistance, T1DM is typically characterized by immune-mediated destruction of insulin-producing pancreatic β cells [2]. The resulting insulin deficiency in T1DM is accompanied by hyperglycemia, altered lipid metabolism, oxidative stress, and endothelial cell dysfunction, leading to metabolic disturbances in various organs, including skeletal muscle [3, 4]. Skeletal muscle is one of the main sites of glucose uptake and regulation of glucose homeostasis. The disturbances in insulin secretion and function in DM disrupt normal carbohydrate, protein, and fatty acid metabolism in skeletal muscles [5, 6]. In T1DM, a significant disturbance in muscle protein turnover, including decreased protein synthesis and increased protein breakdown, has been reported [1, 7]. While skeletal muscles may undergo functional and structural adaptations to metabolic disturbances, the full impact of T1DM on the muscle phenotype and function in humans remains unclear. Still, it is thought to ultimately lead to decreased muscle mass, regeneration capacity, and physical performance [8].

Skeletal muscle fibers are classified based on the expression of different isoforms of myosin heavy chain (MyHC), with each type having particular biochemical and physiological properties related to insulin sensitivity and glucose uptake capacity [9]. Type 1 skeletal muscle fibers are slow-twitch fibers that are highly sensitive to insulin and have a large glucose uptake capacity. Type 2a and 2x skeletal fibers have intermediate insulin sensitivity and glucose uptake capacity, while type 2b fibers have the lowest insulin sensitivity and ability to uptake glucose [10–12]. Changes in the fiber-type composition suggest alterations to substrate metabolism, and such shifts in skeletal muscle fiber-type composition and metabolism have been linked to the pathogenesis of T1DM complications [13]. Studies in T1DM rats and mice have reported degeneration, necrosis, and atrophy of muscle fibers, particularly affecting types 2a and 2b, along with changes in the mitochondrial structure and function [14, 15]. Typical findings in rodent animal models show a shift toward a more oxidative fiber-type composition in T1DM [15, 16], although some human studies have reported an enhanced glycolytic metabolism and a transition to predominantly glycolytic or fast-twitch muscle fibers [17].

Given that some therapeutic approaches that improve the insulin sensitivity of skeletal muscle have been shown to improve glycaemic control and potentially decelerate T1DM disease progression [18, 19], skeletal muscle changes in T1DM have recently become a focal point of both pathophysiological and therapeutic research [13]. The most widely used animal model for studying T1DM myopathy is the streptozotocin (STZ)-induced diabetic rodent model. STZ is a dose-dependent alkylating agent absorbed by pancreatic β-cells, leading to deoxynucleic acid damage, cell death, and subsequent insulin deficiency [20, 21]. Due to its pancreatic cytotoxic effect in various animal species, STZ is widely employed to induce T1DM in rats and mice, providing a cost-effective model that mimics human T1DM [1, 22, 23]. The essential indices of skeletal muscle histochemical and morphologic phenotype, including fiber-type composition, fiber size, intramyocellular lipid content, capillary-to-fiber ratio, and contractile properties, have all been shown to be altered in T1DM [15, 24–26], but considerable inconsistencies exist in findings and interpretations, probably due to methodological heterogeneity. In particular, capillarization parameters are traditionally assessed with two-dimensional (2D) stereological methods, but it has been shown that this conventional approach is remarkably unreliable for quantifying the complex capillary network in skeletal muscles [27–30]. Specific indices, such as structural anisotropy, orientation of linear structures, and topological properties, can be more efficiently and precisely quantified using three-dimensional (3D) analytic methods [27]. Accordingly, the present study aimed to comprehensively analyze the microanatomical changes in the slow-twitch soleus muscle and fast-twitch gluteus maximus and gastrocnemius muscles of STZ-induced diabetic C57BL/6J-OlaHsd mice, including MyHC isoforms expression, and capillary network architecture using a 3D analytic approach.

## Materials and methods

### Animal care and study group protocols

The study was performed with female C57BL/6J-OlaHsd mice (*n* ═ 24) obtained at the age of six weeks from the Harlan Laboratories–Envigo, Italy. Upon acquisition, the animals were reared at the Centre for Laboratory Animals of the Biotechnical Faculty of the University of Ljubljana in individually ventilated cages (IVC system) under controlled conditions of temperature (23 ± 1 ^∘^C), humidity (40%–60%), and 12-h light/dark cycle. The laboratory diet (4RF18, Mucedola, Milan, Italy) and water were available ad libitum during the experiment.

After a seven-day acclimation period, the mice were randomly divided into two study groups: (a) STZ-induced diabetic mice (*n* ═ 12) and (b) non-diabetic mice (*n* ═ 12). T1DM was induced by a single intraperitoneal administration of STZ (200 mg/kg body weight) at eight weeks of age [31, 32]. The STZ-induced model was chosen as an established and commonly employed model for studying T1DM in rodents, and the high dose of STZ was employed to overcome the known resistance of female mice to STZ-induced DM. The non-diabetic mice were treated similarly and received saline instead of STZ. Diabetes was confirmed by determining fasting glucose levels with the Bayer Contour glucose meter (Ascensia Diabetes Care Holdings AG, Switzerland) three weeks after STZ injection administration.

Animals with fasting glucose levels above 25 mmol/L were considered diabetic, whereas those with fasting glucose levels below 8 mmol/L were classified as non-diabetic [33]. Those with glycemic levels between 8–25 mmol/L were therefore not considered for the study. The diabetic mice received no insulin treatment. Since it has been demonstrated that the resulting DM in STZ-induced T1DM female C57BL/6J-OlaHsd mice could be maintained for at least eight months [32], we performed no further measurements of glucose levels after confirming the establishment of DM.

The mice were weighed once a day during the experiment. Six weeks after the STZ application, the animals were sacrificed by cervical dislocation followed by decapitation and bleeding out. Promptly after euthanasia, the gluteus maximus, gastrocnemius, and soleus muscles were harvested from the left hind limb, frozen in liquid nitrogen, and stored at −80 ^∘^C until the cross-sections were cut. Note that the sample for this study (*n* ═ 24) was drawn from another larger study (*n* ═ 36) investigating peripheral neuropathy in mice, in which local anesthetic agents were locally infiltrated in one limb [34]. The included mice were those which survived the previous experiments, met the criteria for glycemic status for the control and diabetic groups, and had no evident damage to the muscles of interest. The muscles were harvested from the contralateral limb without local anesthetic treatment.

### Myosin heavy-chains analysis

#### Cryosection preparation and labeling

Serial transverse 10-µm thick cryosections, prepared from the left gluteus maximus, gastrocnemius, and soleus muscles of each mouse, were marked with four monoclonal antibodies: BA-D5, BF-F3, SC71, and 6H1. Antibodies BA-D5 immunoreactive to MyHC-1 at a dilution of 1:100, antibodies SC71 immunoreactive to MyHC-2a at a dilution of 1:100, and antibodies BF-F3 immunoreactive to MyHC-2b at a dilution of 1:30 were purchased from Deutsche Sammlung von Mikroorganismen und Zellkulturen (DSMZ, Braunschweig, Germany) [35]. These antibodies were detected by a secondary antibody, P0260 (Dako, Glostrup, Denmark), while the antibody 6H1 at a dilution of 1:100 was used for the detection of MyHC-2x/d using the Polymer Detection System (Novolink, Leica Biosystems, Newcastle, UK) [36]. The phenotyping of muscle fibers was based on the expression of MyHC isoforms on serial sections using the indirect immunoperoxidase method as previously described [9, 37].

#### Acquisition and analysis of myosin heavy chains

Serial sections stained with specific monoclonal antibodies for MyHC isoforms were analyzed using a Nikon Eclipse 8000 microscope at ×20 magnification. A Nikon DMX 1200F digitized camera and Lucia GF version 4.82 software (Laboratory Imaging, Prague, Czech Republic) were employed for image acquisition at a 2560 × 1920 pixels resolution. For each muscle (gluteus maximus, gastrocnemius, and soleus muscle) of an individual mouse, three randomly selected fields were analyzed using the image analysis program Ellipse (ViDiTo, Kosice, Slovakia), which allowed delineation and classification of muscle fibers with the estimation of their average diameter and numerical density. Fibers were divided into pure fibers expressing either MyHC-1, MyHC-2a, MyHC-2x, or MyHC-2b and hybrid fibers co-expressing MyHC-1/2×, MyHC-2a/2× or MyHC-2b/2×. A single-blinded evaluator performed the analysis.

### Capillary network analysis

#### Cryosection preparation and labeling

The capillary network was analyzed on 100-µm thick cross-sections of the gluteus maximus of each mouse. A mixture of 7% formaldehyde and 0.1% glutaraldehyde was used to fix the samples. The sections were then exposed to an antigen retrieval solution containing Proteinase K 0.2% (Fermentas, Waltham, MA, USA) for 5 min at 37 ^∘^C. To visualize the basal lamina, we incubated the samples overnight with a rabbit polyclonal antibody against collagen IV at a dilution of 1:200 (ab6586; Abcam, Cambridge, UK). For endothelial cell detection, we used the secondary antibody Alexa Fluor 594 A-11012 at a dilution of 1:100 (Invitrogen, Waltham, MA, USA) and fluorescein-labeled *Grifonia (Bandeiraea) simplicifolia* lectin I at a dilution of 1:300 (FL -1101; Vector Laboratories, Burlingame, CA, USA) [38]. All sections were mounted in ProLong Gold Antifade (Molecular Probes from Life Technologies, Waltham, MA, USA).

#### Acquisition and analysis of capillary networks

Transverse cryosections were analyzed using a Leica SP8 two-channel confocal microscope (Leica Microsystems, Wetzlar, Germany) with an oil immersion objective at ×40 magnification and LAS X 3.5.2.18963 software. Five fields of view were randomly selected for each gluteus maximus muscle of the individual mouse. The gluteus maximus muscle was chosen as 3D capillary network analysis requires larger samples, thus, the soleus and gastrocnemius were inappropriate [27]. Images were acquired as 12-bit images with a spacing of 1 µm at a resolution of 512×512 pixels and a pixel size of 0.618 µm × 0.618 µm. The excitation wavelengths used were 496 nm for the argon laser and 543 nm for the white light laser (pulsed at 78 MHz and tunable from 470–670 nm). The lambda scans showed green and red emission peaks at 546 and 613 nm, respectively. A system of acousto-optical beam splitter, prism-based dispersion, and mirrors was used to filter the emission signal. A specific channel selection band was chosen to prevent crosstalk between the channels. The images of the two channels were taken one after the other. The analysis of the images was performed with the image analysis program Ellipse (ViDiTo, Kosice, Slovakia). The detailed description of the procedure is based on the methodology of Eržen et al. and has been described in detail in previously published articles [10, 34, 39]. The estimated capillary length was expressed as the sum of the lengths of the line segments in the geometric diagram within a fiber neighborhood of 5 µm and defined per muscle fiber length (LL), surface area (LS), and volume (LV) as previously described [40]. Tortuosity (T) was expressed as the sum of the exterior angles (in radians) between successive line segments per total capillary length.

### Ethical statement

The study was conducted in strict compliance with the recommendations of the National Institutes of Health’s Guide for the Care and the Use of Laboratory Animals. All research protocols were reviewed and approved by the Veterinary Administration of the Republic of Slovenia (approval number: 34401-54/2012/5). All experiments were performed in accordance with the European Convention for the Protection of Vertebrate Animals used for Experimental and Other Scientific Purposes (ETS 123) and the National Institutes of Health’s Guide for the Care and the Use of Laboratory Animals.

### Statistical analysis

Statistical analysis was performed using GraphPad Prism 10 (GraphPad Software Inc., San Diego, CA, USA). The Shapiro–Wilk test was used to test the characteristics under study for normality and *F*-test was used to test for equal variance. The independent samples *t*-test was employed to compare T1DM and non-diabetic mice. Data are presented as mean ± standard deviation (SD). Differences were deemed statistically significant at *P* < 0.05.

## Results

The initial weight of the mice and blood glucose levels were similar in both groups and, therefore, did not differ significantly between the STZ-induced T1DM and non-diabetic groups. During the experiment, STZ-induced T1DM mice had significantly higher glucose levels (*P* < 0.0001) and lower average body mass (*P* < 0.0001) than non-diabetic mice (Table 1).

**Table 1:** The comparison of weight and glucose measurements between T1DM mice (*n* ═ 12) and non-diabetic mice (*n* ═ 12)

	**T1DM**	**Non-diabetic**	***P* value**
Weight (day 0) (g)	25.03 ± 1.00	25.39 ± 1.09	ns
Weight (week 5) (g)	19.57 ± 2.06	27.99 ± 1.57	< 0.0001
Glucose (day 0) (mM)	9.21 ± 0.72	9.22 ± 0.99	ns
Glucose (week 3) (mM)	30.19 ± 5.84	10.12 ± 1.00	< 0.0001

Data are presented as mean value ± standard deviation. T1DM: Type 1 diabetes mellitus; ns: Not significant.

**Figure 1.**
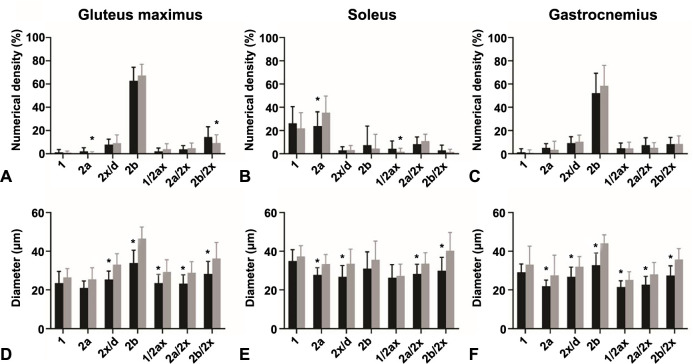
**Numerical density and diameter of (A, D) gluteus maximus, (B, E) soleus, and (C, F) gastrocnemius muscle fibers.** Comparison between type 1 diabetes mellitus mice (black columns; *n* ═ 12) and non-diabetic mice (gray columns; *n* ═ 12). Data are presented as mean ± standard deviation. *P < 0.05.

**Figure 2.**
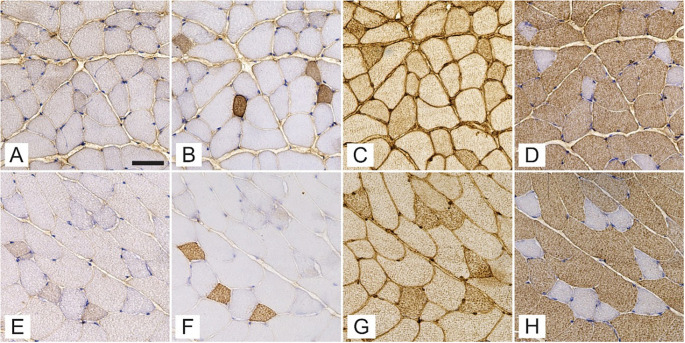
**Expression of myosin heavy chain isoforms 1 (A, E), 2a (B, F), 2x/d (C, G), and 2b (D, H) in successive cross-sections of gluteus maximus muscle of streptozotocin-induced diabetic mice (A–D) and age-matched non-diabetic mice (E–H).** The scale bar indicates 50 µm.

**Figure 3.**
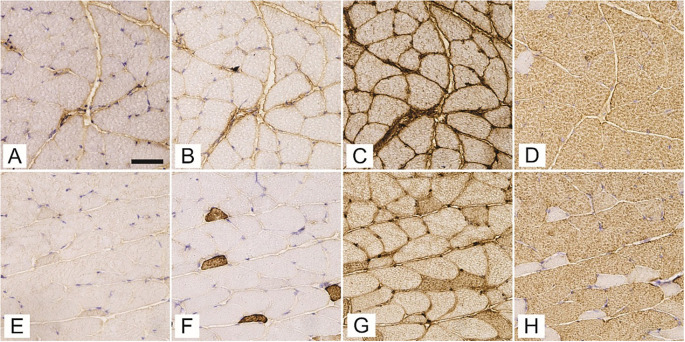
**Expression of myosin heavy chain isoforms 1 (A, E), 2a (B, F), 2x/d (C, G), and 2b (D, H) in successive cross-sections of gastrocnemius muscle of streptozotocin-induced diabetic mice (A–D) and age-matched non-diabetic mice (E–H).**The scale bar indicates 50 µm.

**Figure 4.**
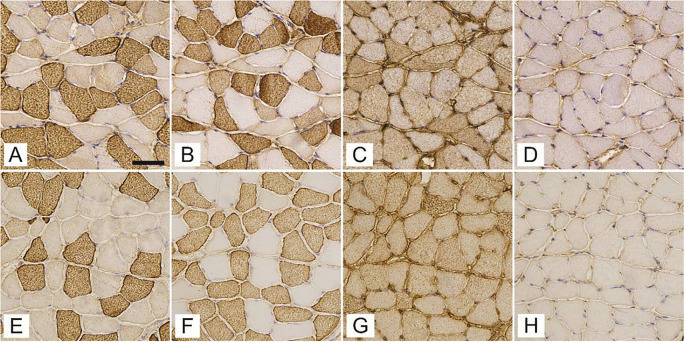
**Expression of myosin heavy chain isoforms 1 (A, E), 2a (B, F), 2x/d (C, G) and 2b (D, H) in successive cross sections of soleus muscle of streptozotocin-induced diabetic mice (A–D) and age-matched non-diabetic mice (E–H).** The scale bar indicates 50 µm.

A total of 2066 and 1799 gluteus maximus muscle fibers, 2196 and 1790 gastrocnemius muscle fibers, and 2386 and 2102 soleus muscle fibers from T1DM and non-diabetic mice, respectively, were assessed for the expression of MyHCs (Figure 1). The gluteus maximus (Figure 2) and gastrocnemius muscles (Figure 3) were predominantly composed of fast-twitch type 2b fibers with no statistically significant differences between T1DM and non-diabetic mice. The soleus muscle was composed mainly of type 2a fibers with a significantly greater proportion in non-diabetic mice (35.6 ± 14.3% vs 23.9 ± 12.1%, *P* ═ 0.03), and type 1 muscle fibers, with no significant differences in numerical densities between diabetic and non-diabetic mice (Figure 4). The numerical proportions of muscle fibers expressing different MyHC isoforms are depicted more precisely in Figure 1.

In the gastrocnemius muscle, the average diameters of muscle fibers 2a (*P* ═ 0.02), 2x/d (*P* ═ 0.001), 2b (*P* ═ 0.001), and the hybrid types (*P* < 0.01) differed significantly between the assessed groups. The diameters of the gluteus maximus fibers were significantly larger in the non-diabetic group for the types 2x/d (*P* ═ 0.001), 2b (*P* ═ 0.001), and hybrid types (*P* ═ 0.001). In the soleus muscle, the diameter of the fibers in the control group, again, showed a larger diameter in fibers 2a (*P* ═ 0.001), 2x/d (*P* ═ 0.001), 2a/x (*P* ═ 0.001), and 2b/2x (*P* < 0.001).

**Figure 5.**
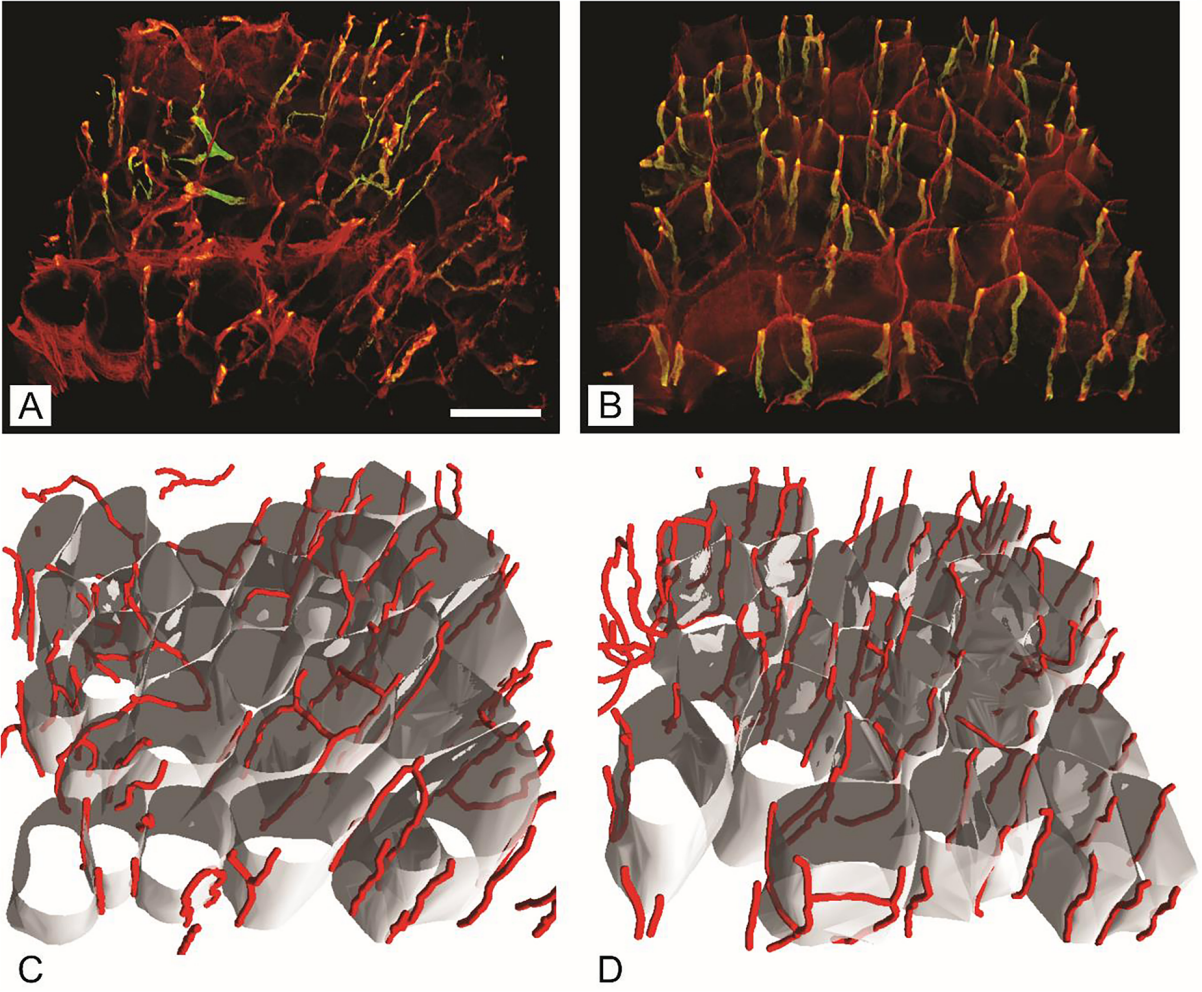
**Capillaries and muscle fibers in gluteus maximus muscle of streptozotocin-induced diabetic mice (B, D) and age-matched non-diabetic mice (A, C).** (A and B) Immunofluorescent staining with volume rendering of capillaries; (C and D) Reconstructed muscle fibers with supplying capillaries. The scale bar indicates 50 µm.

The gluteus maximus capillary network analysis showed statistically significant differences between T1DM and non-diabetic mice in capillary length per muscle fiber volume (*P* < 0.05), with higher values in diabetic mice (Figure 5). The analysis confirmed a larger diameter of muscle fibers in the control group (*P* ═ 0.026), however, there were no statistically significant differences between the groups in capillary length per surface or length of muscle fiber and tortuosity (Table 2).

**Table 2:** The comparison of the capillary distribution of the gluteus maximus muscle between T1DM mice (*n* ═ 12) and non-diabetic mice (*n* ═ 12)

	**T1DM**	**Non-diabetic**	***P* value**
D (µm)	40.93 ± 6.88	47.47 ± 4.35	0.026
LL	2.66 ± 2.52	2.52 ± 0.32	ns
LS (µm^−1^ × 10^−2^)	1.93 ± 0.37	1.66 ± 0.34	ns
LV (µm^−1^ × 10^−3^)	2.19 ± 0.53	1.68 ± 0.48	0.049
Tortuosity (µm^−1^ × 10^−2^)	6.85 ± 1.10	6.86 ± 1.26	ns

Data are presented as mean value ± standard deviation. T1DM: Mice with type 1 diabetes mellitus; D: Diameter of muscle fiber; LL: Length of capillaries per length of muscle fiber; LS: Length of capillaries per surface area of muscle fiber; LV: Length of capillaries per volume of muscle fiber; Tortuosity: The sum of external angles between successive line segments per total length of capillaries (in radians); ns: Not significant.

## Discussion

The present study analyzed the changes in MyHC fiber composition, fiber diameter, and capillary network architecture of slow- and fast-twitch skeletal muscles in STZ-induced T1DM mice. We found that fast-twitch muscles in diabetic and non-diabetic mice were predominantly composed of type 2b fibers, with no significant differences between the groups. However, the slow-twitch soleus muscle of non-diabetic mice had more type 2a fibers but a similar type 1 fiber density to diabetic mice. Remarkably, T1DM mice had smaller fiber diameters and showed increased capillary length per muscle volume, potentially reflecting muscle fiber atrophy.

Quantitative assessment of morphometric parameters of skeletal muscle in STZ-diabetic animals remains scarce [1, 22]. Nevertheless, available studies examining various muscles in STZ-diabetic mice and rats consistently documented a decrease in the proportion and size of muscle fibers [1, 7, 22, 23, 34], consistent with our findings. Skeletal muscle atrophy in DM is intricate and appears to involve several interrelated mechanisms, including disruptions in metabolism, alteration in vascular structure, low serum insulin-associated decline in contractile protein synthesis, and degeneration of motor endplates [41]. Several molecular studies have demonstrated altered expression of muscle atrophy-related genes in STZ-induced diabetes models [42]. In T1DM, diminished insulin/insulin-like growth factor 1 (IGF-1) action in muscles hinders complex I-driven mitochondrial respiration and supercomplex assembly via FoxO-mediated complex I subunit inhibition [43]. Consequently, this disruption impairs mitochondrial function, ultimately triggering skeletal muscle atrophy by upregulating muscle atrophy-related genes like MAFbx and MuRF1 through heightened FoxO transcriptional activity. In addition, DNA microarray analysis has shown that genes controlling proteolytic systems are upregulated in STZ-induced rodent models [44]. The significant decline in body mass in STZ-induced diabetic mice is most likely a consequence of the observed muscle atrophy, given that skeletal muscles constitute roughly 50% of total body mass. Meanwhile, it is possible that T1DM may also induce changes in the number of fibers in addition to a decrease in the diameter of individual skeletal muscle fibers. It has been shown that in long-standing STZ-induced T1DM, more significant muscle fiber atrophy was evident in all fiber types of diabetic fast-twitch muscles compared to the muscle fibers in slower muscles characterized by a higher oxidative metabolism [45].

The most abundant MyHC fibers in healthy, predominantly slow-twitch soleus muscle were type 2a and shifted toward other hybrid fiber types in diabetic mice. These observations are consistent with the results of histochemical and morphological analyses, showing degeneration and atrophy of type 2a and 2b myofibers in the hindlimb muscles of mice and rats subjected to STZ-induced diabetes [1, 14, 15]. Although the proportion of 2a fibers was decreased in the diabetic soleus muscle, other reports suggest that 2a fibers exhibit enhanced adaptability and resilience against pathological alterations [16]. Meanwhile, our finding of significant differences between the groups in the MyHC fiber type composition of the soleus but not the gluteus maximus and gastrocnemius muscles mirrors previous findings that noticeable shifts in muscle fiber phenotype are particularly pronounced in predominantly slow-twitch muscles in obesity and insulin resistance [46]. In contrast, Klueber et al. [15] reported a notable reduction in the size of both type 2a and type 2b fibers in the fast-twitch extensor digitorum longus of STZ-induced diabetic Swiss Webster and C57BL/IKsJ db/db mice, but with a relatively more pronounced decrease in mean fiber area of the type 2b fibers [15].

The density and distribution of capillaries around muscle fibers predict skeletal muscle oxygenation and metabolic capacity. Initial studies on humans with T1DM showed no change in capillary density [34], however, more recent research in T1DM mice revealed decreased expression of pro-angiogenesis-related genes and increased expression of anti-angiogenic genes [47]. Notable capillary regression accompanied by a reduction in the vascular endothelial growth factor-A/thrombospondin-1 ratio was reported in the soleus and plantaris muscles of BioBreeding T1DM rats, reflecting an altered skeletal muscle angio-adaptive environment [48]. Similarly, research in obesity and other insulin-resistant phenotypes suggests a decline in the capillary network of skeletal muscles and impaired insulin-mediated capillary recruitment, both leading to insulin resistance and impairing glucose uptake [49]. This consideration is remarkable, given the increasing recognition of the role of insulin resistance in T1DM [3]. However, our 3D analysis of the capillary network of the gluteus maximus muscle demonstrated higher values of the capillary length per muscle fiber volume in T1DM mice. It is noteworthy that the determination of capillary length is also contingent upon the methodology used for sampling, image capture, segmentation, and measurement, and the relationship between capillary length and fiber volume is influenced by various factors, including muscle and fiber typology, metabolic demands, and physiological adaptations [39]. Although we found no differences in capillary tortuosity between the groups, STZ-induced DM has been shown to result in less tortuous capillaries and diminished capillary diameters [50]. In general, skeletal muscle capillary tortuosity exhibits a curvilinear rise as sarcomere length decreases, compensating for the decrease in capillary density as the cross-sectional area of muscle fibers increases [51].

We postulate that the higher values of the capillary length per muscle fiber volume in T1DM mice may be due to the reduced diameter of muscle fibers and may not necessarily reflect an actual angiogenic increase in capillarity. This is supported by the fact that the volume-independent parameter capillary length per muscle fiber length was unaffected, whereas the volume-dependent capillary length per muscle fiber volume was significantly greater in the T1DM group. In addition, T1DM has been shown to result in more pronounced atrophy of all fiber types of fast-twitch muscles such as gluteus maximus. It is plausible that the increased capillary length per muscle fiber volume may be less evident in slow-twitch muscles where fiber atrophy is less pronounced. Hence, future studies should include both slow- and fast-twitch muscles and analyze the capillary indices according to specific fiber types [45]. Conversely, it has also been reported that skeletal muscle capillary distribution primarily adjusts in proportion to fiber size and remains relatively unaffected by fiber type or oxidative potential [40, 52]. Our previous study with the same 3D approach in obese, insulin-resistant mice showed increased capillary density around small muscle fibers (diameter < 40 µm), reflected in a higher volume-insensitive capillary length per muscle fiber length ratio, which may be a compensatory mechanism related to impaired glucose metabolism in obese mice [10]. On the other hand, Krause et al. [23] observed a decreased capillary-to-fiber ratio in gastrocnemius and plantaris muscle of STZ-induced mice. This ratio is commonly used for 2D assessment of muscle fiber capillary network and is comparable to capillary length per muscle fiber length.

Although conventional 2D stereological techniques are technically less cumbersome and time consuming, when evaluating the complex microvasculature, the 3D techniques are considered superior to 2D methods [27, 28]. However, the majority of existing data on capillary network changes in DM are based on 2D analyses of tissue cross-sections with diverse indices for capillarization assessment, leading to significant disparities between studies [53]. Skeletal muscle capillaries create an intricate and interconnected network, the arrangement of which is greatly influenced by the geometry of the muscle fibers. Using the traditional approach of counting capillary profiles from 2D images of thin transverse sections may substantially underestimate the capillary length and other indices characterizing muscle capillary pattern. Our 3D reconstruction technique, which has been rigorously validated in various species, incorporates both muscle fiber size-related and size-independent indices to account for variations in fiber size and interstitial tissue, reducing capillary supply estimation error by up to 75% compared to the 2D method [28, 30, 40]. Accordingly, our approach enables a more robust evaluation and interpretation of the capillary configurational changes in muscle tissues. The findings with the 3D analysis of capillary indices underscore the need for cautious interpretation of 2D data on capillary changes in skeletal muscles. For example, variations in muscle fiber dimensions between experimental and control animals stemming from muscle atrophy or growth inhibition had led to the erroneous inference of increased capillary density due to capillary proliferation in the past, whereas in reality, only alterations in fiber size might have occurred [51, 54].

Furthermore, it is noteworthy that while the 3D analytical method employed in our study is suited for stacks of images acquired by confocal microscopy, 3D visualization and quantification of tissue microvascular indices may potentially be realized with other advanced microscopy techniques, such as ultramicroscopy, micro-computed tomography (micro-CT), multiphoton microscopy, and serial block-face scanning electron microscopy [55–58]. Notably, ultramicroscopy was shown to be particularly valuable for imaging and quantifying capillary networks, providing 3D information superior to histological assessment [58]. Further studies are needed to demonstrate the advantages and limitations of the various 3D techniques in characterizing skeletal muscle capillarity.

We acknowledge a few limitations of our study. First, the study exclusively utilized female mice, therefore, future investigations on male mice are imperative, as sex differences affect insulin sensitivity in rodents [59, 60]. Nevertheless, while most preclinical studies on T1DM complications have predominantly utilized male mice due to the reported resistance of female mice to diabetogenic agents like STZ, it was recently shown that in female C57BL/6J-OlaHsd mice, such resistance to T1DM induction can be simply overcome by increasing the dose of STZ administered [32]. Accordingly, in the present study, we sought to contribute to bridging the current gap in the literature by investigating female mice, using a higher dose of STZ for T1DM induction. Second, the capillary network analysis was conducted solely on the gluteus maximus muscle because 3D analysis of capillaries requires large samples [27], and capillary indices were assessed without discrimination by fiber type. It has been shown that utilizing indices specific to fiber types enhances the capillary data analysis in skeletal muscle samples [61]. Third, given that diabetic neuropathy results in diminished muscle cross-sectional area [15, 19, 62], the relationship between T1DM neuropathic changes and fiber composition, morphometry, and capillarization may be explored in future studies.

Finally, although studies on the impact of T1DM on skeletal muscle predominantly use STZ-induced diabetic rodents, the independent effects of STZ on skeletal muscle may complicate the characterization of diabetic myopathy. This may be particularly significant in the context of the relatively higher dose of STZ required to induce T1DM in female mice. STZ attenuates muscle fiber growth in vivo [63] and can lead to decreased infiltration of monocytes and macrophages that mediate muscle regeneration [64]. In addition, despite similarities in hyperglycemia/hyperinsulinemia, distinctive contractile, metabolic, and phenotypic properties were observed between skeletal muscles of STZ and *Ins2^Akita+/−^* (a genetic model of T1DM) diabetic mice, with STZ mice having notably larger type I fibers and more intramyocellular lipid than *Ins2^Akita+/−^* and control mice [23]. Furthermore, the exact timing of the induction of capillarization remodeling in STZ-induced DM is not well established. Nevertheless, as previous studies have reported histologically evident hyperglycemia-related fiber structural and capillariztaion changes at 4–5 weeks after STZ treatment [45], [47], we consider that our analysis of the tissues at six weeks post-STZ treatment is a reasonable duration to observe T1DM-attributable fiber and capillary network changes. On the other hand, sustaining a hyperglycaemic state over an extended duration proves challenging in STZ-induced diabetic animal models, making it complex to investigate the effects of prolonged disease duration and concurrent age-related alterations [14]. These findings and considerations question the suitability of the STZ-induced model in representing the multifactorial phenotype of human T1DM.

## Conclusion

In this study, we investigated the impact of STZ-induced T1DM on the morphological characteristics of slow- and fast-twitch skeletal muscles, including MyHC fiber composition, structure, and capillary network architecture. In T1DM and non-diabetic mice, the fast-twitch gluteus maximus and gastrocnemius muscles were primarily composed of fast-twitch type 2b fibers, with no significant differences. On the other hand, the slow-twitch soleus muscle in non-diabetic mice exhibited a greater proportion of type 2a fibers but comparable type 1 fiber densities to diabetic mice. In addition, reduced fiber diameters were noted in T1DM mice, and 3D assessment of the capillary network revealed increased capillary length per muscle volume in the gluteus maximus of diabetic mice compared to the control group. However, there were no notable differences between the groups regarding capillary length per fiber length, surface area, and capillary tortuosity. The relative increase in capillarization of skeletal muscle fibers in STZ-induced T1DM mice model is possibly due to changes in muscle fiber diameter, and this should be taken into consideration when interpreting experimental results.

Our findings further underscore the capacity of a 3D analytical method to provide a more detailed characterization of skeletal muscle capillary architecture and emphasize the need for cautious interpretation of 2D data on capillary configurational changes in skeletal muscles in T1DM. Further investigations should characterize the capillary morphology according to individual fiber types and address potential sex-based differences and the suitability of the STZ model for representing the human T1DM skeletal muscle fiber and capillary phenotype.

## Data Availability

The datasets used and analyzed during the present study are available from the corresponding author upon reasonable request.

## References

[1] Aughsteen AA, Billah Khair AM, Suleiman AA (2006). Quantitative morphometric study of the skeletal muscles of normal and streptozotocin-diabetic rats. J Pancreas [Internet].

[2] Ndisang JF, Vannacci A, Rastogi S (2017). Insulin resistance, type 1 and type 2 diabetes, and related complications 2017. J Diabetes Res.

[3] Kaul K, Apostolopoulou M, Roden M (2015). Insulin resistance in type 1 diabetes mellitus. Metab Clin Exp.

[4] Katsarou A, Gudbjörnsdottir S, Rawshani A, Dabelea D, Bonifacio E, Anderson BJ (2017 Mar 30). Type 1 diabetes mellitus. Nat Rev Dis Primers.

[5] Fazakerley DJ, Krycer JR, Kearney AL, Hocking SL, James DE (2019 Oct). Muscle and adipose tissue insulin resistance: malady without mechanism?. J Lipid Res.

[6] Nutter CA, Jaworski E, Verma SK, Perez-Carrasco Y, Kuyumcu-Martinez MN (2017 Oct 17). Developmentally regulated alternative splicing is perturbed in type 1 diabetic skeletal muscle. Muscle Nerve.

[7] Hegarty P V, Rosholt MN (1981). Effects of streptozotocin-induced diabetes on the number and diameter of fibres in different skeletal muscles of the rat. J Anat.

[8] Almeida S, Riddell MC, Cafarelli E (2008 Feb). Slower conduction velocity and motor unit discharge frequency are associated with muscle fatigue during isometric exercise in type 1 diabetes mellitus. Muscle Nerve.

[9] Cvetko E, Karen P, Eržen I (2012 Sep). Myosin heavy chain composition of the human sternocleidomastoid muscle. Ann Anat.

[10] Umek N, Horvat S, Cvetko E (2021 Oct 22). Skeletal muscle and fiber type-specific intramyocellular lipid accumulation in obese mice. Bosn J Basic Med Sci.

[11] Idris I, Gray S, Donnely R (2006 Jan 24). Insulin action in skeletal muscle. Ann N Y Acad Sci [Internet].

[12] Lefaucheur L (2010 Feb). A second look into fibre typing—Relation to meat quality. Meat Sci.

[13] Coleman SK (2015). Skeletal muscle as a therapeutic target for delaying type 1 diabetic complications. World J Diabetes.

[14] Ozaki K, Matsuura T, Narama I (2001 Sep). Histochemical and morphometrical analysis of skeletal muscle in spontaneous diabetic WBN/Kob rat. Acta Neuropathol.

[15] Klueber KM, Feczko JD (1994 May). Ultrastructural, histochemical, and morphometric analysis of skeletal muscle in a murine model of type I diabetes. Anat Rec.

[16] Armstrong RB, Gollnick PD, Ianuzzo CD (1975 Oct). Histochemical properties of skeletal muscle fibers in streptozotocin-diabetic rats. Cell Tissue Res.

[17] Fritzsche K, Blüher M, Schering S, Buchwalow I, Kern M, Linke A (2008 May 9). Metabolic profile and nitric oxide synthase expression of skeletal muscle fibers are altered in patients with type 1 diabetes. Exp Clin Endocrinol Diabetes.

[18] D’Souza DM, Al-Sajee D, Hawke TJ (2013 Dec). Diabetic myopathy: impact of diabetes mellitus on skeletal muscle progenitor cells. Front Physiol.

[19] Andersen H, Gadeberg PC, Brock B, Jakobsen J (1997 Aug 19). Muscular atrophy in diabetic neuropathy: a stereological magnetic resonance imaging study. Diabetologia.

[20] Junod A, Lambert AE, Stauffacher W, Renold AE (1969 Nov 1). Diabetogenic action of streptozotocin: relationship of dose to metabolic response. J Clin Invest.

[21] Elsner M, Guldbakke B, Tiedge M, Munday R, Lenzen S (2000 Nov 30). Relative importance of transport and alkylation for pancreatic beta-cell toxicity of streptozotocin. Diabetologia.

[22] Fahim MA, el-Sabban F, Davidson N (1998 Jun). Muscle contractility decrement and correlated morphology during the pathogenesis of streptozotocin-diabetic mice. Anat Rec.

[23] Krause MP, Riddell MC, Gordon CS, Abdullah S, Cafarelli E, Hawke TJ (2009). Diabetic myopathy differs between Ins2 Akita/ and streptozotocin-induced type 1 diabetic models. J Appl Physiol [Internet].

[24] Tamaki T, Muramatsu K, Ikutomo M, Oshiro N, Hayashi H, Niwa M (2018 Sep 6). Effects of streptozotocin-induced diabetes on leg muscle contractile properties and motor neuron morphology in rats. Anat Sci Int.

[25] Chao TT, Ianuzzo CD, Armstrong RB, Albright JT, Anapolle SE (1976 May). Ultrastructural alterations in skeletal muscle fibers of streptozotocin-diabetic rats. Cell Tissue Res.

[26] Sexton WL, Poole DC, Mathieu-Costello O (1994 Apr 1). Microcirculatory structure-function relationships in skeletal muscle of diabetic rats. Amer J Physiol Heart Circ Physiol.

[27] Umek N, Janáček J, Cvetko E, Eržen I (2021 May 24). Horizontal deformation of skeletal muscle thick sections visualised by confocal microscopy. J Microsc.

[28] Čebašek V, Eržen I, Vyhnal A, Janáček J, Ribarič S, Kubínová L (2010 Jan). The estimation error of skeletal muscle capillary supply is significantly reduced by 3D method. Microvasc Res.

[29] Schaad L, Hlushchuk R, Barré S, Gianni-Barrera R, Haberthür D, Banfi A (2017 Feb 7). Correlative imaging of the murine hind limb vasculature and muscle tissue by MicroCT and light microscopy. Sci Rep.

[30] Eržen I, Janáček J, Kubinová L (2011 Feb 28). Characterization of the capillary network in skeletal muscles from 3D data. Physiol Res.

[31] Furman BL (2021 Apr 27). Streptozotocin-induced diabetic models in mice and rats. Curr Protoc.

[32] Saadane A, Lessieur EM, Du Y, Liu H, Kern TS (2020 Sep 17). Successful induction of diabetes in mice demonstrates no gender difference in development of early diabetic retinopathy. PLoS One.

[33] Pan H, Ding Y, Yan N, Nie Y, Li M, Tong L (2018 Sep). Trehalose prevents sciatic nerve damage to and apoptosis of Schwann cells of streptozotocin-induced diabetic C57BL/6J mice. Biomed Pharmacother.

[34] Markova L, Umek N, Horvat S, Hadžić A, Kuroda M, Pintarič TS (2020 Jul 17). Neurotoxicity of bupivacaine and liposome bupivacaine after sciatic nerve block in healthy and streptozotocin-induced diabetic mice. BMC Vet Res.

[35] Schiaffino S, Gorza L, Sartore S, Saggin L, Ausoni S, Vianello M (1989 Jun). Three myosin heavy chain isoforms in type 2 skeletal muscle fibres. J Muscle Res Cell Motil.

[36] Lucas CA, Kang LHD, Hoh JFY (2000 May). Monospecific antibodies against the three mammalian fast limb myosin heavy chains. Biochem Biophys Res Commun.

[37] Karen P, Števanec M, Smerdu V, Cvetko E, Kubínová L, Eržen I (2009 Aug 17). Software for muscle fibre type classification and analysis. Eur J Histochem.

[38] Laitinen L (1987 Apr). Griffonia simplicifolia lectins bind specifically to endothelial cells and some epithelial cells in mouse tissues. Histochem J.

[39] Eržen I, Janáček J, Kreft M, Kubínová L, Cvetko E (2018 Jan 2). Capillary Network morphometry of pig soleus muscle significantly changes in 24 hours after death. J Histochem Cytochem.

[40] Janáček J, Čebašek V, Kubínová L, Ribarič S, Eržen I (2009 May 5). 3D visualization and measurement of capillaries supplying metabolically different fiber types in the rat extensor digitorum longus muscle during denervation and reinnervation. J Histochem Cytochem.

[41] Jun L, Robinson M, Geetha T, Broderick TL, Babu JR (2023 Feb 3). Prevalence and mechanisms of skeletal muscle atrophy in metabolic conditions. Int J Mol Sci.

[42] Shen Y, Li M, Wang K, Qi G, Liu H, Wang W (2022 Jun 30). Diabetic muscular atrophy: molecular mechanisms and promising therapies. Front Endocrinol (Lausanne).

[43] Bhardwaj G, Penniman CM, Jena J, Suarez Beltran PA, Foster C, Poro K (2021 Sep 15). Insulin and IGF-1 receptors regulate complex I–dependent mitochondrial bioenergetics and supercomplexes via FoxOs in muscle. J Clin Invest.

[44] Saliu TP, Kumrungsee T, Miyata K, Tominaga H, Yazawa N, Hashimoto K (2022 Jan). Comparative study on molecular mechanism of diabetic myopathy in two different types of streptozotocin-induced diabetic models. Life Sci.

[45] Jerković R, Bosnar A, Jurisić-Erzen D, Azman J, Starcević-Klasan G, Peharec S (2009). The effects of long-term experimental diabetes mellitus type I on skeletal muscle regeneration capacity. Coll Antropol.

[46] Torgan CE, Brozinick JT, Kastello GM, Ivy JL (1989 Nov 1). Muscle morphological and biochemical adaptations to training in obese Zucker rats. J Appl Physiol.

[47] Kivelä R, Silvennoinen M, Touvra AM, Maarit Lehti T, Kainulainen H, Vihko V (2006 Jul). Effects of experimental type 1 diabetes and exercise training on angiogenic gene expression and capillarization in skeletal muscle. FASEB J.

[48] Aiken J, Mandel ER, Riddell MC, Birot O (2019 Jan 26). Hyperglycaemia correlates with skeletal muscle capillary regression and is associated with alterations in the murine double minute-2/forkhead box O1/thrombospondin-1 pathway in type 1 diabetic BioBreeding rats. Diab Vasc Dis Res.

[49] Gomes JLP, Fernandes T, Soci UPR, Silveira AC, Barretti DLM, Negrão CE (2017). Obesity downregulates MicroRNA-126 inducing capillary rarefaction in skeletal muscle: effects of aerobic exercise training. Oxid Med Cell Longev.

[50] Kindig CA, Sexton WL, Fedde MR, Poole DC (1998 Feb). Skeletal muscle microcirculatory structure and hemodynamics in diabetes. Respir Physiol.

[51] Poole DC, Batra S, Mathieu-Costello O, Rakusan K (1992 Apr). Capillary geometrical changes with fiber shortening in rat myocardium. Circ Res.

[52] Ahmed S, Egginton S, Jakeman P, Mannion A, Ross H (1997 Jan 1). Is human skeletal muscle capillary supply modelled according to fibre size or fibre type?. Exp Physiol.

[53] Montero D (2016 Mar 1). Comment on Prior et al. Increased skeletal muscle capillarization independently enhances insulin sensitivity in older adults after exercise training and detraining. Diabetes.

[54] Cassin S, Gilbert R, Bunnell C, Johnson E (1971 Feb 1). Capillary development during exposure to chronic hypoxia. Am J Physiol Legacy Content.

[55] Christensen DJ, Nedergaard M.

[56] Buchacker T, Mühlfeld C, Wrede C, Wagner WL, Beare R, McCormick M (2019 Nov 21). Assessment of the alveolar capillary network in the postnatal mouse lung in 3D using serial block-face scanning electron microscopy. Front Physiol.

[57] Schneider B, Kopf KW, Mason E, Dawson M, Coronado Escobar D, Majka SM (2023 Jul 27). Microcomputed tomography visualization and quantitation of the pulmonary arterial microvascular tree in mouse models of chronic lung disease. Pulm Circ.

[58] Epah J, Pálfi K, Dienst FL, Malacarne PF, Bremer R, Salamon M (2018). 3D imaging and quantitative analysis of vascular networks: a comparison of ultramicroscopy and micro-computed tomography. Theranostics.

[59] Lundsgaard AM, Kiens B (2014 Nov 13). Gender differences in skeletal muscle substrate metabolism—Molecular mechanisms and insulin sensitivity. Front Endocrinol (Lausanne).

[60] Green S, Kiely C, O’Connor E, Gildea N, O’Shea D, Egaña M (2022 Jan). Differential effects of sex on adaptive responses of skeletal muscle vasodilation to exercise training in type 2 diabetes. J Diabetes Complications.

[61] Čebašek V, Ribarič S (2019 Aug 16). Changes in local capillarity of pure and hybrid MyHC muscle fiber types after nerve injury in rat extensor digitorum longus muscle (EDL). Histochem Cell Biol.

[62] Andreassen CS, Jakobsen J, Ringgaard S, Ejskjaer N, Andersen H (2009 Jun 12). Accelerated atrophy of lower leg and foot muscles—A follow-up study of long-term diabetic polyneuropathy using magnetic resonance imaging (MRI). Diabetologia.

[63] Johnston APW, Campbell JE, Found JG, Riddell MC, Hawke TJ (2007 Mar). Streptozotocin induces G_2_ arrest in skeletal muscle myoblasts and impairs muscle growth in vivo. Am J Physiol Cell Physiol.

[64] Hidmark AS, Nawroth PP, Fleming T (2017 May 15). STZ causes depletion of immune cells in sciatic nerve and dorsal root ganglion in experimental diabetes. J Neuroimmunol.

